# Tuning the Transdermal Delivery of Hydroquinone upon Formulation with Novel Permeation Enhancers

**DOI:** 10.3390/pharmaceutics11040167

**Published:** 2019-04-04

**Authors:** Dolores R. Serrano, María José Gordo, Antonio Matji, Salvador González, Aikaterini Lalatsa, Juan José Torrado

**Affiliations:** 1 Department of Pharmaceutics and Food Technology, School of Pharmacy, Complutense University of Madrid, Ramón y Cajal square, 28040 Madrid, Spain; mgordo03@ucm.es (M.J.G.); Antonio.matji@farmaciash24.com (A.M.); 2 University Institute of Industrial Pharmacy, Complutense University, 28040 Madrid, Spain; 3 Department of Medicine and Medical Specialties, Alcalá University, 28040 Madrid, Spain; salvagonrod@gmail.com; 4 School of Pharmacy and Biomedical Sciences, University of Portsmouth, St. Michael’s Building, White Swan Road, Portsmouth PO1 2DT, UK; katerina.lalatsa@port.ac.uk

**Keywords:** hydroquinone, transdermal delivery, Franz cells, permeability enhancers, stability, multivariate analysis

## Abstract

Hydroquinone (HQ) is an anti-hyperpigmentation agent with poor physicochemical stability. HQ formulations are currently elaborated by compounding in local pharmacies. Variability in the characteristics of HQ topical formulations can lead to remarkable differences in terms of their stability, efficacy, and toxicity. Four different semisolid O/W formulations with 5% HQ were prepared using: (i) Beeler´s base plus antioxidants (F1), (ii) Beeler´s base and dimethyl isosorbide (DMI) as solubiliser (F2), (iii) olive oil and DMI (F3), and (iv) Nourivan^®^, a skin-moisturising and antioxidant base, along with DMI (F4). Amongst the four formulations, F3 showed the greatest physicochemical stability with less tendency to coalescence but with marked chromatic aberrations. An inverse correlation was established by multivariate analysis between the mean droplet size in volume and the steady-state flux, which explains why F3, with the smallest droplet size and the most hydrophobic excipients, exhibited the highest permeation across both types of membranes with enhancement ratios of 2.26 and 5.67-fold across Strat-M^®^ and mouse skin, respectively, compared to F1. It is crucial to understand how the HQ is formulated, bearing in mind that the use of different excipients can tune the transdermal delivery of HQ significantly.

## 1. Introduction

Currently, hydroquinone (HQ) ointments and creams are one of the most frequent types of formulations prescribed by dermatologists to treat the hyperpigmentation of the skin [1]. HQ is used as an anti-hyperpigmentation agent in skin-lightening formulations at different strengths up to 10% (*w*/*w*), although the most common concentration ranges between 4–5% (*w*/*w*) [2,3]. The poor physicochemical stability of HQ topical formulations is a crucial problem that has led to low interest within the pharmaceutical industry for the manufacturing of HQ topical formulations; thus, patients need to rely on extemporaneously produced products prepared by community pharmacies. The chemical instability of HQ can lead to the formation of p-benzoquinone (pBQ), which is carcinogenic, and the production of other compounds that lead to dark chromatic aberrations [4,5,6]. The hydrophilic nature of HQ is an extra challenge in developing topical formulations, as its permeability is limited unless an appropriate penetration enhancer is included in the extemporaneous prepared topical products [7,8,9]. However, if HQ is not localised in the skin, it can lead to severe adverse effects due to its systemic absorption such as hepatotoxicity, nephrotoxicity, and neurobehavioural alterations [10,11]. Thus, variability in the physicochemical characteristics of different HQ formulations can lead to remarkable differences in terms of its stability, efficacy, and toxicity, and it is an interesting topic of research due to its significant clinical implications.

The hypothesis underpinning this work is that the transdermal permeation enhancers utilised in the manufacturing of topical HQ formulations may have a significant effect on both the stability and the permeability (efficacy versus toxicity) across the skin, which should be taken into consideration by dermatologists when prescribing. Thus, we aim to compare the physicochemical stability and permeability of four different HQ topical formulations with a dose strength of 5% using a variety of permeation enhancers commonly employed in the extemporaneous compounding of HQ. Four different semisolid oil in water (O/W) formulations (coded as F1–F4, see Table 1) were prepared. F1 was manufactured using the conventional Beeler´s base (which is an anionic o/w emulsion containing sodium lauryl sulphate as surfactant, Acofarma^®^ 2018 [12]). Beeler’s base has been selected as a reference formulation, as currently, the preparation of HQ topical formulations by Spanish community pharmacies is based on this base. Antioxidant agents such as vitamin C and E were also incorporated to reduce HQ oxidation. F2 to F4 included dimethyl isosorbide (DMI) instead of propylene glycol, as it has been reported to be a suitable permeation enhancer for HQ with less toxicity [13]. Beeler´s base was also utilised in the preparation of F2, whereas an olive oil base supplement consisting mostly of oleic acid was used in the preparation of F3, and Nourivan^®^ base was employed in the preparation of F4. Nourivan^®^ base was selected due to its skin-moisturising and antioxidant properties, which is of special importance in the extemporaneous dispensing of drugs susceptible to oxidation such as HQ (Fagron^®^, 2018) [14]. The conventional pH for hydroquinone topical formulations is preferably acidic, and generally below or close to a pH of 4, even though this can be harsh to the skin and possibly other components of the product. Hydroquinone is more stable, and less likely to discolour under acidic conditions. Variations in pH have been shown to result in marked discoloration [5,15].

## 2. Materials and Methods

### 2.1. Materials

HQ USP quality was donated from Cantabria Laboratories (Madrid, Spain). pBQ (>98%) was supplied by Sigma-Aldrich (Madrid, Spain). Beeler’s base was supplied by Acofarma (Madrid, Spain), while Nourivan^®^ was supplied by Fagron Ibérica (Madrid, Spain). All of the excipients were of pharmacopoeia grade.

### 2.2. Preparation of HQ Formulations (F1–F4)

The composition of the four O/W cream formulations is illustrated in Table 1. Briefly, HQ was finely grounded and then either dispersed in propylene glycol (PG) or dissolved in DMI using a mortar and pestle. Then, the corresponding base (Beeler´s base, olive oil, or Nourivan^®^) was incorporated and mixed in the mortar and pestle. Dissolved Vitamin C (Vit. C) and E (Vit. E) were added in F1 at the end of the process. An Unguator mixer (Microcaya, Bilbao, Spain) was employed between 3–5 min in order to ensure a homogenous semisolid mixture. Once prepared, the final pH was measured and reported in Table 1.

### 2.3. Physicochemical Characterisation of HQ Formulations

A particle size analyser (Microtrac S3500, Microtrac, PA, USA) was used for determination of the mean particle size and particle size distribution of HQ formulations. The size and distribution of the prepared formulations were expressed by the volume median diameter (MV) and the D10, D50, and D90 (indicating the percentages of particles having 10%, 50%, and 90% of the diameter equal to or lower than the given value). The span was calculated as a measure of polydispersity using the following equation:

Span = (D90 − D10)/D50(1)

Prior to measurement, HQ formulation samples were diluted (1/100) with deionised water. Each sample was measured in triplicate [3,16].

The rheological behavior of the formulations was evaluated in triplicate using a Brookfield (Middleborough, MA, USA) Model DV-III fitted with a temperature control probe. A 5-cm cone–plate measuring geometry was used. The temperature of all the measurements was maintained at 25 °C. Viscosity (cP) and shear stress (D × cm^−2^) were determined over a speed rate from 0 to 15 rpm, and a shear rate from 0 to 30 (1/s). Prior to measurements, a standard of 311.25 Pa·s was analysed [17].

Apparent water content (%) was evaluated as weight loss. The loss of mass was measured by an infrared balance (Mettler PM100 and LP16, Columbus, OH, USA). The final point was set up to 120 s without weight loss and heated at 105 °C.

### 2.4. Physicochemical Stability Studies

The physical stability of HQ formulations including size and size distribution was evaluated at different time points after storage at three different temperatures: 6 °C, 25 °C, and 40 °C. Chemical stability was also analysed at the same time by high-performance liquid chromatography (HPLC). HQ formulations (0.1 g) were dispersed in 10 mL of deionised water followed by 10 min of bath sonication and centrifugation (8000 rpm, 10 min). Supernatants were filtered by 0.45 µm using a hydrophilic polytetrafluoroethylene (PTFE) filter (Millipore Millex-LCR^®^, Billerica, MA, USA) and subsequently diluted 1 in 100 with mobile phase and analysed by HPLC (see method below described) for HQ and p-benzoquinone (pBQ) quantification. The degradation rate of HQ formulations was calculated by fitting the percentage of HQ degraded at different time points to a first-order reaction. The Arrhenius equation was used in order to estimate the activation energy and the effect of temperature on the degradation rates of HQ (Equation (2)).

(2) $$K=Ae^{\frac{-E_{a}}{RT}}$$

where *K* is the degradation rate constant of HQ (% degraded/day), A is the collision factor, *T* is the absolute temperature in Kelvin, R is the gas constant (1.985 cal/mol/K), and Ea is the activation energy in cal/mol.

### 2.5. In Vitro Permeation Studies Using Strat-M® Membranes

Strat-M^®^ membranes (Millipore, Billerica, MA, USA) were mounted between the donor and receptor chamber of Franz diffusion cells (Soham Scientific, Soham, UK) with an effective diffusion area of 1.76 cm^2^ and a cell volume of 12 ml filled with freshly phosphate-buffered solution (PBS, pH 7.4). The diffusion cells were maintained at 37 ± 0.5 °C, and the fluid in the receptor chambers was stirred continuously at 600 rpm. Accurately weighed (200 mg) and prepared HQ formulations were loaded in the donor chambers and spread over a thin layer on the Strat-M^®^ membrane. Samples (1 mL) from the receptor chambers, at several time intervals (5 min, 10 min, 15 min, 30 min, 60 min, 120 min, 180 min, 240 min, 360 min, and 480 min) were withdrawn for HPLC determination without further dilution, and the volume was replaced immediately with fresh phosphate buffer solution to keep sink conditions. The cumulative amounts of HQ that permeated through the Strat-M^®^ membrane were plotted as a function of time [3]. Each formulation was tested in triplicate. Regression analysis was used to calculate the slopes and intercepts of the linear portion of each graph. The following equation (Equation (3)) was applied to each formulation to calculate the steady-state flux:

(3) $$Jss=\frac{dC}{dX}\times A$$

where *Jss* is the steady-state flux (μg/cm^2^/h), d*C*/d*X* is the amount of HQ permeating the membrane over time (μg/h), and A is the surface area of contact of the formulation [18]. The permeability coefficient (*P*) was calculated by using Equation (4):

*P* = *Jss*/*cd* (4)

where *cd* is amount of drug applied in the donor compartment (200 mg of formulation equivalent to 10 mg of HQ). The diffusion coefficient was calculated by using the following equation:

(5) $$Jss=\frac{D\times K}{h}\;cd$$

where h is the thickness of the membrane. The enhancement ratio (ER) was calculated as the ratio of steady-state transdermal flux from each formulation compared to Formulation (1) [19].

### 2.6. In Vitro Permeation Studies using Healthy Mouse Skin

Mouse skin was removed from a male-bred NMRI (8 weeks, 25 g) that had been previously euthanised according to the Ethical Committee Regulations of the University, Community of Madrid PROEX 041/18 (27/4/2018). The skin was placed flat in foil and frozen within a sealed plastic bag in the freezer (−20 °C), and was used within a month. The skin was shaved and suspended in 60 °C deionised water for 30 seconds, after which the underlying muscle tissue and hypodermis were manually removed. Skin was thawed in PBS pH 7.4 for 20 min prior to being mounted between the donor and receptor chamber of a Franz diffusion cell. Experiments were performed in quadruplicate, and a similar methodology was utilised when artificial skin membranes (Strat M^®^ membrane, Millipore, Billerica, MA, USA) were used.

### 2.7. Quantification of Drug Amount Trapped in Mouse Skin after In Vitro Permeability Assay

Following each permeability study (6 h), the skin samples were wiped with an ethanol-impregnated cotton bud to remove the excess of formulation. Samples were cut in half. One half was fixed using 4% paraformaldehyde (pH 7) and placed in the fridge for histological studies. The other half was weighed and homogenised with 2 mL of PBS pH 7.4 buffer. A 1:2 dilution with methanol was performed. The mixture was vortexed (2 min) and then centrifuged (10 min, 10,000 rpm). The supernatant was transferred into an HPLC vial and analysed for drug content using the HPLC method described below.

### 2.8. HPLC Quantification for HQ and pBQ

HQ and pBQ were isocratically eluted using a Hichrom (Reading, UK) Partisil 10 ODS C18 reverse-phase column (200 × 4.6 mm, 5 μm) and a mobile phase that consisted of a monobasic phosphate buffer pH 3: methanol (95:5 *v*/*v*) with a flow rate of 1.5 mL/min. Absorbance was monitored at 289 nm for HQ and 252 nm for pBQ. The injection volume was set at 20 µL and 100 µL for stability and permeation studies, respectively. The retention time of HQ was 3.1 min, and it was 5.2 min for pBQ. A linear regression calibration curve between 0.1–50 µg/mL was obtained to extrapolate the concentration values.

### 2.9. Mechanistic Release Models

The permeability data of HQ from each formulation was fitted using different mathematical models in order to investigate the mechanism involved in the HQ release from the different formulations [20]: zero order (Equation (6)), first order (Equation (7)), Hixson–Crowell (Equation (8)), Higuchi (Equation (9)), and Korsmeyer–Peppas (Equation (10)):

(6) $$Q_{t}=Q_{0}+K_{0}t$$

(7) $$\ln Q_{t}=\ln Q_{0}+K_{1}t$$

(8) $$Q_{0}^{1/3}-Q_{t}^{1/3}=K_{2}t$$

(9) $$Q_{t}=K_{H}\sqrt{t}$$

(10) $$\frac{Q_{t}}{Q_{\infty }}=K_{H}t^{n}$$

where *Q_t_* is the amount of drug dissolved in time *t*, *Q*_0_ is the initial amount of drug in the solution (most times, *Q*_0_ = 0), Q_∞_ is the initial amount of drug in the formulation; *Q_t_*/*Q_∞_* is the fraction of drug release at time *t*; *K*_0_ is the zero-order release constant, *K_1_* is the first-order release constant, *Ks* is a constant incorporating the surface–volume relation; *K_H_* is the Higuchi order release constant, *K_KP_* is a constant that describes the structural and geometric characteristics of the drug dosage form, and *n* is the release exponent that describes the drug release mechanism. The *n* can have a value of 0.5, 0.45, or 0.43 when the particle shape is a thin film, a cylinder, or a sphere, respectively, which corresponds to Fickian release controlled by diffusion. Anomalous non-Fickian transport is described when n is between those values and 1 (0.5 < *n* < 1 for thin films, 0.45 < *n*< 1 for cylinders, and 0.43 < *n* < 1 for spheres). *n* = 1 corresponds with zero-order release [21]. To test the applicability of the drug release model, the regression coefficient (*R*^2^) was calculated [22].

### 2.10. Skin Histological Analysis after Exposure to HQ Formulations

Fixed mouse skin samples with 4% paraformaldehyde exposed to HQ formulations and healthy untreated NMRI skin samples were placed in Leica biopsy cassettes, and then dehydrated using a tissue processor (Shandon, Citadel 2000 Tissue Processor, Thermo Scientific, Basingstoke, UK). The dehydration process involved tissues automatically placed first in deionised water for 2 min followed by ascending concentrations of ethanol (2 h at 50%, 2 h at 75%, 2 h at 90%, and 2 h × 3 in 100% ethanol) and finally into Histoclear medium for 4 h (histological cleaning agent, Agar scientific, Stansted, UK) prior to paraffin wax embedding (3 h).

Once the samples had been dehydrated following preparation, they were placed in metal moulds (Tissue Tek Stainless Steel Base Moulds for Uni-Cassettes and Process/Embedding Systems) and filled with preheated, melted paraffin wax (Histosec paraffin wax pastilles, Merck Millipore, Billerica, MA, USA) using a wax embedder (Leica EG1150 H Heated Paraffin Embedding Module, Wetzlar, Germany). Then, the filled moulds were placed on a cold plate (Leica EG1150 C Cold Plate Module) and once the paraffin had set, they were removed from the metal moulds and kept on the cold plate ready for use.

The paraffin-embedded mouse skin samples were sectioned using a microtome (Leica RM-2235 Microtome) attached to a 10-μm microtome blade (Leica Disposable Blade, high profile (818) (80 mm long, 14 mm high). Finely cut sections of samples were floated on a 40 °C water bath (Leica HI1210 Water bath) to flatten the sections out. Then, the cut sections were placed on microscope slides (Shandon Colorfrost Plus Microscope slides, Thermo Scientific, Basingstoke, UK), dried on a hot plate (Leica HI1220 Flattening table, Leica Biosistems, Newcastle upon Tyne, UK) at 37 °C, and finally stained with haematoxylin and eosin (H&E).

Slides were placed on a hot plate (Leica HI1220 Flattening table, Leica Biosistems, Newcastle upon Tyne, UK) at 60 °C for 15 min to allow the paraffin wax to melt using the IHC World for H&E staining method (IHC World, 2018) [23]. Then, heated slides were immersed in xylene (two sets, 10 min in each) to completely remove residual paraffin wax, followed by treatment in two sets of ethanol (both 100%) for 5 min each to dehydrate the samples. Slides were placed in a staining rack and coated with haematoxylin (0.1%) for 10 min, and then rinsed under tap water for 5 min. Excess haematoxylin was removed by applying acid alcohol (1% HCl 0.02 M in 70% Ethanol) for 5 seconds; then, slides were rinsed under tap water. Slides were immersed in Eosin (1% solution) for 5 min; then, slides were rinsed under tap water for 1 to 5 min. Stained specimen samples were placed in deionised water for 3 min, followed by ascending grades of ethanol (50%, 70%, 95%) for 3 min each up to 100% ethanol for 6 min each for dehydration. Samples were placed into xylene for 6 min and then mounted in DPX mountant media and covered with glass microscope slide cover slips (22 × 22 mm, Menzel-Gläser, Braunschweig, Germany). The stained mouse skin sample slides were observed under a light microscope with an attached camera (GMX-L1500BHTG Biological, Trinocular Microscope with GXCAM-1.3, GT Vision Ltd, Stansfield, UK). Images were captured, saved, and analysed by using GXCapture7 software.

### 2.11. Statistical Analysis

Statistical analyses were performed via one-way ANOVA test using Minitab 16 (Minitab Ltd., Coventry, U.K.) followed by Tukey’s test. Statistical significance was set at *p* < 0.05. A multivariate data analysis was performed using The Unscrambler^®^ X software (CAMO Software, Norway). Eight variables (mean size in volume, span, viscosity, water content, stability at 25 °C and 40 °C at day 30 expressed as percentage of remaining HQ, steady-state flux across mouse skin and Strat-M^®^) were analysed by principal component analysis (PCA). PCA was employed to study the systematic variability and the relationships between variables and scores (the four HQ formulations). The correlation loadings of the PCs were represented to understand the variance for each variable for a given PC, giving information about the source of the variability inside the dataset [24].

## 3. Results and Discussion

### 3.1. Characterisation of HQ Formulations

The droplet size, mean viscosity, and water content of the four HQ formulations freshly prepared are illustrated in Table 2. The trend in droplet size expressed in volume, number, or area was the same in all the cases, in which F1 was the formulation with the highest droplet size (61.8 ± 3.4 µm) followed by F2 (44.9 ± 4.5 µm), F4 (26.0 ± 1.9 µm), and F3 (8.3 ± 0.8 µm). In contrast, the polydispersity index showed that F3 and F4, even though they exhibited the smallest droplet size, had a twofold higher span compared to F1 and F2. Similar results were obtained for the viscosity: F3 and F4 exhibited higher viscosity values than F1 and F2 at 15 rpm. Interestingly, the apparent water content of F1, F2, and F4 formulations was close to the real water content (50–70%) after preparation, while for F3, there was an important discrepancy, bearing in mind that the true water content was 50.8% while only 16.8% was detected. This discrepancy can be related to F3 being capable of retaining water molecules in its physicochemical structure even at 105 °C, which probably can lead to a greater emollient activity than the other three formulations.

**Figure 1:**
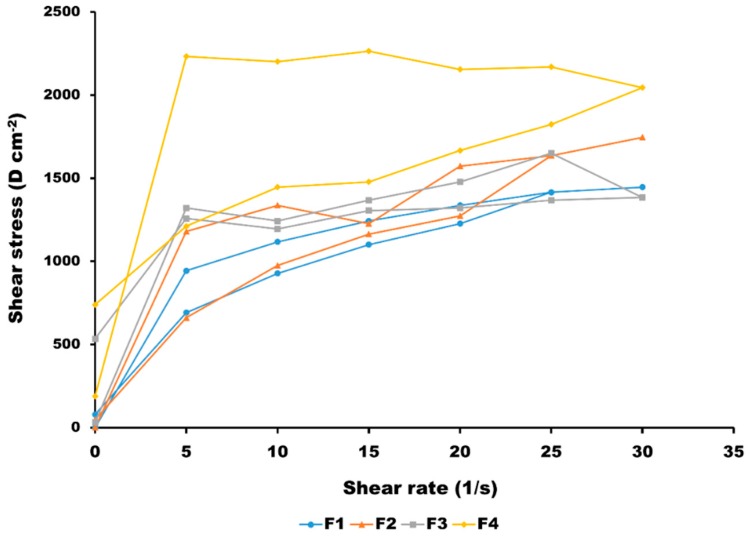
Rheograms of the four HQ formulations (*n* = 3).

Figure 1 shows the rheological behaviour of the four HQ formulations after stress from 0 to 15 rpm. Even though all of the emulsions exhibited a pseudoplastic behaviour and thixotropy, it is important to understand how differences in droplet size and polydispersity affect the different rheological properties amongst the formulations, as this impacts on the required force for the application of the formulation on the skin. A pseudoplastic behaviour is quite common in creams and emulsions, because the orientation of the particles changes to align with the direction of flow. The original orientation is restored over a period of time, after which the external force is removed. Unlike Newtonian fluids, the viscosity of pseudoplastic materials increases with decreasing droplet size, as shown for F3 and F4 versus F1 and F2. The flow behaviour in highly concentrated emulsions is governed by the total interfacial area. A change in droplet size at a constant volume fraction causes a horizontal shift in the viscosity versus the shear rate [25]. The thixotropy was more evident in F4, which probably is related to the high polydispersity of the formulation in terms of particle size, and hence, the longer time required for the particles to rearrange to its initial conformation. F4 also showed to some extent a Bingham pseudoplastic behaviour requiring a higher shear stress at low shear rates for the flow to start. This may be related to the ability of the different droplet sizes to exhibit a closer packing compared to uniform and larger droplet sizes, as in the case of F1 or F2.

### 3.2. Physical and Chemical Stability

**Figure 2:**
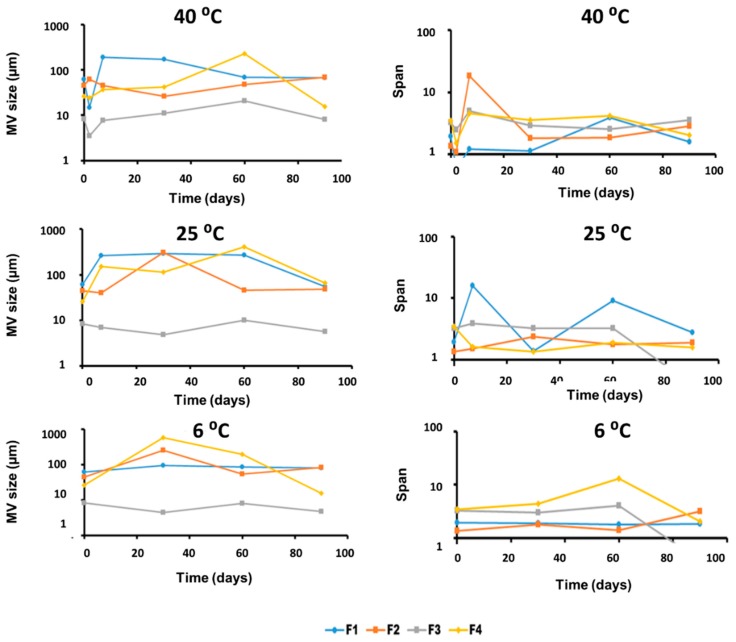
Evolution of MV size (**left**) and polydispersity of size expressed as span (**right**) of samples stored at different temperatures in semilogarithmic scale.

**Figure 3:**
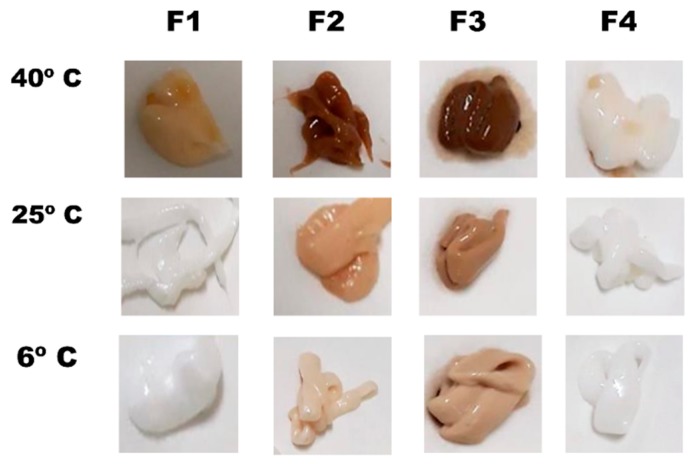
Chromatic aberrations on formulations stores during 30 days at different temperatures.

Physical stability was studied as the evolution of the mean volume size and span values of samples stored at different temperatures over a three-month period (Figure 2). The most physically stable formulation was F3, whereas F1, F2, and F4 showed a significant increase in the particle size over time, and a greater span probably due to a coalescence process. The increase in particle size was more prominent at higher temperatures. The chemical stability of HQ formulations was studied based on the following three parameters: chromatic aberrations, HQ content (%), and p-BQ content (%). Figure 3 shows the differences among formulations in terms of chromatic aberrations. It is clearly observed that F3 was the most affected formulation by the chromatic aberrations, which was probably due to its higher oil content, whereas F4 was the less susceptible formulation even after 30 days of storage at 40 °C. The antioxidant capacity of the Nourivan^®^ base is clearly demonstrated in Figure 3.

**Figure 4:**
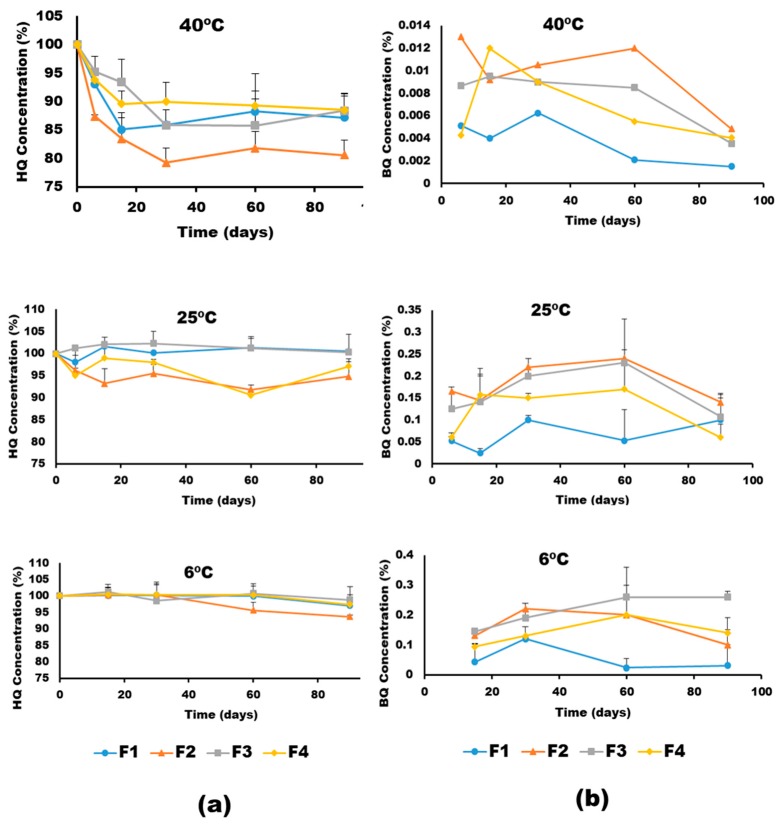
Chemical stability of HQ formulations upon storage at different temperatures expressed as (**a**) HQ degradation and (**b**) p-benzoquinone (pBQ) formation.

Figure 4 show the effect of temperature on the HQ degradation and pBQ formation of the different formulations. All of the formulations were stable (>95% HQ) after 30 days at 6 °C. However, temperature greatly affected the chemical stability of HQ. The degradation rates of HQ calculated by fitting the experimental data to a first-order reaction (*R*^2^ >0.9) showed that F3 was the most stable formulation followed by F4 > F1 > F2 (Table 3). The degradation of HQ at 40 °C occurred at a threefold and 10-fold higher rate than at 25 °C and 6 °C, respectively. The activation energy calculated from the Arrhenius equation was 12.5 ± 2.3 Kcal/mol, which is actually associated with poor chemically stable drugs [26]. Regarding the pBQ (with recommended exposure limits of 0.1 ppm time weighted average, NIOSH 2018) [27], the levels found in the four HQ formulations were below 0.02% in all the conditions, in which F1 was the formulation with the lowest formation of pBQ. The levels of pBQ were reduced over time, which can be related to a secondary degradation of pBQ, and can explain why the values rose up to 30–60 days, but lowered after three months.

### 3.3. In Vitro Permeation Studies and Histological Analysis

**Figure 5:**
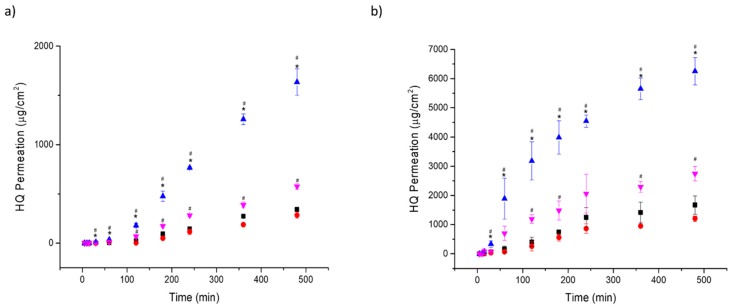
In vitro permeability of HQ formulations across different membranes. (**a**) Strat-M® membrane; (**b**) Mouse skin. Key: -■- Formulation 1; -●- Formulation 2, -▲- Formulation 3; -▼- Formulation 4. # *p*-value < 0.05 compared to Formulation 1 and * *p*-value < 0.05 compared to Formulation 4.

**Figure 6:**
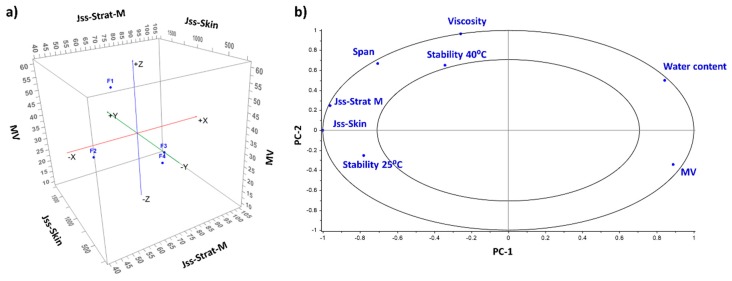
Multivariate analysis of the physicochemical properties of the four HQ formulations and their permeability across mouse skin and Strat-M^®^. (**a**) Three-dimensional (3D) scatter plot comparing MV and Jss across mouse skin and Strat-M^®^; (**b**) Correlation loading plot obtained after principal component analysis (PCA). The average value of the following parameters was included for the MVA analysis: mean size in volume (MV), viscosity, span, water content, stability at 25 °C and 40 °C at 30 days expressed as the percentage remaining of HQ, Jss across mouse skin and Strat-M^®^.

In vitro permeation studies were performed using a synthetic membrane (Strat-M^®^) and mouse skin (Table 4 and Figure 5). F3 exhibited the highest steady-state transdermal flux across both types of membranes. The enhancement ratio was significantly larger (2.26 and 5.67-fold across Strat-M^®^ and mouse skin, respectively) than F1 (*p* < 0.05). Much greater flux was obtained across mouse skin than across Strat-M^®^, which is probably due to the low molecular weight of the HQ (110 g/mol) and its hydrophilicity (log *P* = 0.59), taking into account that the Strat-M^®^ membrane has been validated for drugs that have larger molecular weights than HQ and are more hydrophobic in nature [28,29]. In addition, the shortest lag time was observed with F3, indicating that it had the fastest onset of action (*p* < 0.05). F4 also showed a superior permeation than F1, with an enhancement ratio of 1.58 and 2.27 across Strat-M^®^ and mouse skin, respectively. The better performance of F3 and F4 is associated with their chemical composition as well as their smaller droplet size. F2 exhibited a similar permeability profile than F1, which could be because both of them have a larger droplet size and contain Beeler’s base as their major component. This correlation between particle size and permeation is clearly observed in Figure 6. The PCA correlation loading plot showed that the mean droplet size in volume was inversely correlated with the steady-state flux and the viscosity.

Another factor that can potentially explain the differences in permeability amongst the four formulations is the drug release kinetic (Table 5). The release profiles can be best explained by the Higuchi and Korsmeyer–Peppas models, as the plots show high linearity (*R*^2^ > 0.99). The permeation of the four formulations across Strat-M^®^ followed a Korsmeyer–Peppas release with n values ~ 1, whereas F3 and F4 formulations showed a release profile across mouse skin that is better explained by the Higuchi model compared to F1 and F2 (modelled by the Korsmeyer–Peppas release with an n value of 0.94). The Korsmeyer–Peppas model suggests a two-step release mechanism of the drug embedded in the matrix: polymer relaxation (matrix swelling) followed by diffusion [30]. In those systems with higher lag time, the model that best describes the release is the Korsmeyer–Peppas. In these cases, the lag time is related to the matrix swelling followed by a linear drug release, which corresponds to the secondary diffusion step according to the Korsmeyer–Peppas model. The n value is similar to one, which indicated that the release is also close to a zero-release kinetic. In the case of the permeation of F3 and F4 across the mouse skin, the lag time is negligible, indicating that the diffusion of the drug across the skin starts almost immediately. This ability is probably conferred by the effect of oleic acid in the olive oil base in F3, and the components of Nourivan^®^ in F4. DMI can disrupt the stratum corneum lipid organisation, making it more permeable to HQ. However, DMI by itself does not increase the permeation across the skin (F2 has similar or lower permeability than F1). The combination of DMI with oleic acid and Nourivan^®^ have an enhanced effect on the HQ diffusion coefficient [31,32].

Another difference that can be pointed out amongst the four formulations is the amount of HQ that accumulated in the mouse skin after 6 h. The highest amount of drug was found after the exposure to F1 followed by F2, F4, and F3, which has an inverse relation to the steady-state flux. One of the explanations to justify these results is that F1 and F2 contain mostly Beeler’s base, which is anionic and has a higher potential to interact with the positively charge proteins of the skin (such as filaggrin, a histidine-rich matrix protein of keratinised epidermis [33], and hence, it is retained in the stratum corneum and epidermis in higher concentration instead of passing across [34,35].

**Figure 7:**
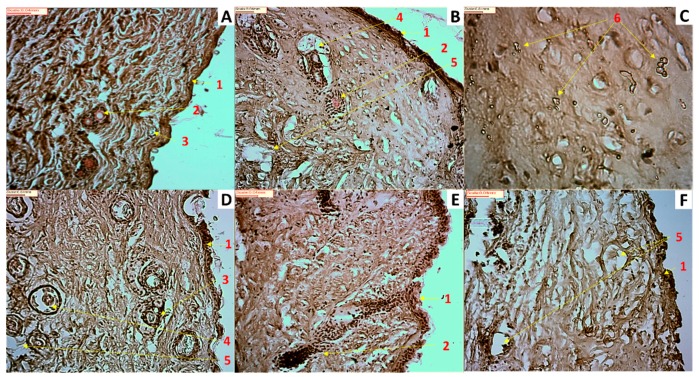
Photomicrographs of untreated and exposed to HQ formulations mouse skin. (**A**) Untreated skin (20× magnification); (**B**) Skin exposed to HQ1 formulation (20× magnification); (**C**) Skin exposed to HQ1 formulation (40× magnification); (**D**) Skin exposed to HQ2 formulation (20× magnification); (**E**) Skin exposed to HQ3 formulation (20× magnification); (**F**) Skin exposed to HQ4 formulation (20× magnification). Key: 1- Stratum corneum, 2- Hair follicle, 3- Sebaceous gland, 4- Possible inflammatory cell, 5- Possible epithelial necrosis, 6- Possible drug depot accumulation.

Figure 7 shows photomicrographs of untreated and exposed to HQ mouse skin. Fig 7 shows some signs of toxicity such as a non-well-defined stratum corneum layer, which could explain the highest permeability across skin compared to Strat-M^®^, as well as the infiltration of some inflammatory cells and epithelial necrosis. It is worthy to note that within the skin exposed to the F1 formulation at greater magnification (40×), some crystalline deposits were observed, which can be attributable to the HQ accumulated in the stratum corneum and epidermis. This can justify the larger amounts of HQ found in the mouse skin after exposure to the F1 formulation. Hence, the cause of low-skin bioavailability for F1 appears to be the reservoir that the drug can form on and in the stratum corneum as well as deposition on the skin. The crystallisation of drugs in skin following topical application has been previously described as being the vehicle employed in the formulation key for this to happen [36].

Previous studies on humans have shown that the dermal absorption of HQ can reach a bioavailability of 45% after 24-h application [37]. The prolonged use of HQ topical products is associated with exogenous ochronosis, and major concerns have been raised due to the carcinogenic potential of HQ reported by animal studies [38,39]. In 2014, the United States Cosmetic Ingredient Review [40] stated that the use of HQ in cosmetic formulations is safe at concentrations below 1% for discontinuous and brief periods. However, in some countries such as Japan, cosmetics can contain up to a 10-fold higher concentration of HQ [41]. In Europe, HQ creams and ointments can be prescribed by dermatologists at different dose strengths up to 10%, and hence, the risk of toxicity and adverse effects raises exponentially. For this reason, it is crucial to understand how the HQ should be formulated, bearing in mind that the use of different permeation enhancers can tune the transdermal delivery of HQ significantly.

One current European cosmetics regulation (Cosmetics Regulation 1223/2009) restricts and precludes the use of HQ in cosmetics. However, dermatologists still need HQ for the treatment of patients with serious hyperpigmentation problems, and typically, high HQ doses are prescribed and used under specialist supervision. Toxicity is related to high permeation that is not limited to the skin. Utilising olive oil or Nourivan^®^ as vehicles in the formulation can enable high local permeation and thus the delivery of lower and most likely safer doses. Until HQ topical medicines are not commercialised by pharmaceutical companies endorsed with well-designed clinical studies, it is important that clinicians should prescribe not only the active ingredient and the dose strength, but also the excipients and the modus operandi for the extemporaneous compounding of HQ formulations in order to limit the risk of variability both in efficacy and toxicity. In our study, we have shown that the substitution of the most common anionic base (Beeler’s Base) employed by Spanish pharmacists during the formulation of HQ topical creams by other alternative excipients such as olive oil or Nourivan^®^ can lead to significantly higher permeation across the skin and hence, lower doses would be required to elicit the same dermatological effect. Otherwise, the risk of toxicity may be exacerbated. Olive oil or Nourivan^®^ have also shown a superior performance in terms of physical and chemical stability compared to those formulations containing Beeler’s base, which can lead to higher patient compliance.

## 4. Conclusions

The physicochemical stability, permeation, and cytotoxicity of four different semisolid O/W formulations with 5% HQ has been evaluated: F1 containing Beeler´s base plus antioxidants, F2 with Beeler´s base and DMI as a solubiliser, F3 formulated with olive oil and DMI, and F4 utilising Nourivan^®^, a skin-moisturising and antioxidant base, along with DMI. The emulsion with the smallest droplet size after dilution was F3 followed by F4 < F2 < F1. Regarding their physicochemical stability, F3 showed the lowest degradation rate of HQ and the smallest variation in droplet size over time; however, F3 exhibited a greater chromatic aberration than the other three formulations. In contrast, F1 had the lowest formation of pBQ, which may be related to the absence of DMI in the formulation.

An inverse correlation was established between the mean droplet size in volume and the steady-state flux, which explains why F3 exhibited the shortest lag time and the highest permeation across both types of membranes with enhancement ratios of 2.26 and 5.67-fold across Strat-M^®^ and mouse skin, respectively, compared to F1. In contrast, the largest amount of HQ found in the skin after 6 h of exposure was F1 followed by F2, which can be justified by the interaction between the cationic proteins in the skin and the anionic nature of the Beeler´s base (sodium lauryl sulphate). Some signs of toxicity were found on the skin after 6 h of exposure with all of the formulations. In conclusion, it is crucial that clinicians prescribe topical HQ formulation indicating not only the dose strength but also the excipients and the modus operandi for the extemporaneous compounding to limit the risk of variability both in efficacy and toxicity.

## Figures and Tables

**Table 1:** Composition of the four developed hydroquinone (HQ) formulations expressed in weight percentage. Key: DMI: Isosorbide dimethyl ether, PG: Propylene glycol, Vit. C: Vitamin C, Vit. E: Vitamin E. Further details in Tables S1 and S2.

Formulation	HQ (%)	PG (%)	Vit. C (%)	Vit E (%)	DMI (%)	Beeler’s Base (%)	Olive Oil Base * (%)	Nourivan^®^ (%)	pH
F1	5	5	1.25	1.25	-	87.5	-	-	3.6
F2	5	-	-	-	15	80	-	-	3.7
F3	5	-	-	-	15	-	80	-	3.6
F4	5	-	-	-	15	-	-	80	3.2

* The base contains 2.5% oleic acid and 1.8% α-tocopherol acetate.

**Table 2:** Mean droplet size expressed in volume (MV), area (MA), and number (MN), span, viscosity, and apparent water content of the different HQ formulations.

Parameters	F1	F2	F3	F4
MV (µm)	61.8	44.9	8.3	26.0
MN (µm)	12.1	15.2	1.0	0.9
MA (µm)	38.4	32.9	3.0	7.3
Span	1.9	1.4	3.2	3.5
Viscosity (Pa·s)	88.8	99.0	113.2	156.6
Apparent water content (%)	60.4	54.8	16.8	69.8

**Table 3:** Degradation constants (percentage of degraded HQ/day) at different storage conditions.

T (°C)	F1	F2	F3	F4
40	0.0107	0.0115	0.0048	0.0072
25	0.0033	0.0046	0.0006	0.005
6	0.001	0.0011	0.0002	0.001

**Table 4:** Comparison of skin permeation of HQ formulations (F) across synthetic (S.M. for Strat-M^®^) or natural (M.S. for Mouse skin) membranes. Key: Jss, steady-state transdermal flux calculated from the slope of the Cartesian plot of the cumulative amount of the drug present in the receptor compartment versus time; ER, enhancement ratio, calculated as the ratio of steady-state transdermal flux from each formulation compared to formulation 1; P, permeability coefficient calculated by using formula *Jss*/*cd* (cd is the amount of drug applied in the donor compartment, so 200 mg of formulation is equivalent to 10 mg of HQ); D, diffusion coefficient (cm^2^/h) calculated by using formula *Jss* = *d.k*/*h* × *cd* (where h is the thickness of the Strat-M^®^ or mouse skin); NA, not applicable.

F	Membrane	Jss (µg/cm^2^/h)	Lag time (h)	P (cm/h) × 10^2^	D (cm^2^/h) × 10^3^	ER	Amount of HQ in the Skin (mg/g)
F1	S.M.	49.3 ± 2.4	0.95 ± 0.06	0.49 ± 0.02	0.54 ± 0.03	-	NA
F2	S.M.	39.3 ± 3.9	1.18 ± 0.09	0.39 ± 0.03	0.43 ± 0.04	0.8 ± 0.1	NA
F3	S.M.	106.3 ± 6.5	0.47 ± 0.08	1.06 ± 0.08	1.17 ± 0.09	2.2 ± 0.2	NA
F4	S.M.	78.1 ± 3.3	0.79 ± 0.07	0.78 ± 0.03	0.85 ± 0.03	1.6 ± 0.1	NA
F1	M.S.	309.9 ± 66.3	0.35 ± 0.08	3.09 ± 0.66	4.31 ± 0.92	-	3.58 ± 1.21
F2	M.S.	221.5 ± 43.1	0.42 ± 0.08	2.21 ± 0.42	3.10 ± 0.06	0.7 ± 0.1	2.50 ± 1.43
F3	M.S.	1754.1 ± 184.9	0.12 ± 0.06	17.5 ± 1.82	24.5 ± 2.58	5.7 ± 0.6	1.36 ± 0.84
F4	M.S.	700.5 ± 213.0	0.12 ± 0.03	7.00 ± 0.21	9.81 ± 0.25	2.3 ± 0.7	1.57 ± 0.78

**Table 5:** Mechanistic release kinetics of HQ from topical formulations (F). Regression coefficient (*R*^2^) values from the permeability data (expressed in %/cm^2^) through synthetic (S.M.) or natural membranes (M.S.) using the following kinetic equations: zero order, first order, Higuchi, Hixson–Crowell and Korsmeyer–Peppas.

F	Membrane	Zero Order	First Order	Higuchi	Hixson–Crowell	Korsmeyer–Peppas
F1	S.M.	0.9851	0.9846	0.9299	0.9848	0.9919
F2	S.M.	0.9735	0.9728	0.9047	0.9731	0.9923
F3	S.M.	0.9919	0.9901	0.9448	0.9908	0.9951
F4	S.M.	0.9923	0.9918	0.9474	0.9919	0.9950
F1	M.S.	0.9797	0.9833	0.9767	0.9822	0.9835
F2	M.S.	0.9801	0.9822	0.9723	0.9815	0.9821
F3	M.S.	0.9517	0.9852	0.9917	0.9774	0.9837
F4	M.S.	0.9653	0.9760	0.9920	0.9728	0.9863

## Supplementary Materials

The following are available online at https://www.mdpi.com/1999-4923/11/4/167/s1, Table S1: Virgin olive oil characteristics. All of the physicochemical tests were conformed with Pharmacopeia requirements. Table S2: Nourivan^®^ characteristics. Composition: Purified water, cetearyl alcohol, polysorbate 60, C13-C16 isoparaffin, C12-C14 isoparaffin, C13-C15 alkane, glyceryl stearate, polyacrylate 13, polyisobutene, polysorbate 20, polyurethane-39, stearyl behenate, cetyl alcohol, ascorbic acid, benzoic acid, sodium bisulfite, sorbic acid, tocopheryl acetate.
