# Supplementary material for: Tuning the Transdermal Delivery of Hydroquinone upon Formulation with Novel Permeation Enhancers
Source: Pharmaceutics. 2019 Apr 4;11(4):167. doi: 10.3390/pharmaceutics11040167 (PMC6523612; doi:10.3390/pharmaceutics11040167)
Supplement: Supplementary file 1 [file pharmaceutics-11-00167-s001.pdf]

pharmaceutics

Supplementary Materials: Tuning the Transdermal Delivery of Hydroquinone upon Formulation with Novel Permeation Enhancers

Dolores R. Serrano, María José Gordo, Antonio Matji, Salvador González, Aikaterini Lalatsa and Juan José Torrado

Table S1. Virgin olive oil characteristics. All physicochemical tests were conformed with Pharmacopeia requirements.

Test	Result	Limit
Acidity	0.1 %	≤ 0.8
Peroxide index	5.8 meq O_2/kg	≤ 20
Humidity and volatile substances	0.09 %	≤ 0.2
Insoluble impurities	< 0.05 %	≤ 0.1
Refractive index	1.468	1.4677–1.4705
Ethyl esters	< 10	Max 35
Content of total sterols	1347 mg/kg	≥ 1000
Cholesterol	0.1 %	≤ 0.5
Miristic acid (C14:0)	< 0.01 %	≤ 0.03

Table S2. Nourivan® characteristics. Composition: Purified water, cetearyl alcohol, polysorbate 60, C13-C16 isoparaffin, C12-C14 isoparaffin, C13-C15 alkane, glyceryl stearate, polyacrylate 13, polyisobutene, polysorbate 20, polyurethane-39, stearyl behenate, cetyl alcohol, ascorbic acid, benzoic acid, sodium bisulfite, sorbic acid, tocopheryl acetate.

Test	Result	Specifications
Identification	Conform	Method A, B (A0133720-28)
Relative density	0.98 g/ml	0.97-1 g/ml
pH	3.5	3.5 - 5.5
Viscosity	172400 mPa s	100000 – 350000 mPa s
Benzoic acid	0.096 %	0.06- 0.12 %
Sorbic acid	0.09 %	0.06- 0.12 %
Total content of aerobic microorganism	< 10 CFU/g	< 1000 CFU/g
Total content of fungi and yeast	< 10CFU/g	< 100 CFU/g
Staphylococcus aureous	Conform	Absence
Pseudomona aeruginosa	Conform	Absence
